# Limy Bile Syndrome: A Report of a Rare Case

**DOI:** 10.7759/cureus.27473

**Published:** 2022-07-30

**Authors:** Nachiket P Rahate, Prashant V Rahate

**Affiliations:** 1 Radiology, Jawaharlal Nehru Medical College, Wardha, IND; 2 Surgery, Seven Star Hospital, Nagpur, IND

**Keywords:** ursodeoxycholic acid, gallbladder, liger function tests, limy bile syndrome, laporoscopic cholecystectomy

## Abstract

Milk of Calcium Bile or Limy Bile Syndrome (LBS) is a sporadic and infrequent complication of cholecystitis in which the gallbladder is filled with radio-opaque, abnormal bile secretion. A 40-year-old female came to a tertiary care hospital with symptoms of recurrent pain upper abdomen for two years. On examination, the patient had mild jaundice and mild tenderness at Murphy’s point exacerbated on deep inspiration. Laboratory investigations suggested raised levels of bilirubin and hepatic enzymes. Upper GI endoscopy revealed a normal GI tract. A hepatobiliary iminodiacetic acid-cholecystokinin (HIDA-CCK) scan suggested a gallbladder ejection fraction of 5%. Cholecystectomy was done the next day. Infrared imaging under Indocyanine green (ICG) dye revealed a completely dark gallbladder. The patient was asked to take ursodeoxycholic acid preparation post-operatively for four weeks. This case of LBS was discharged on the third postoperative day. She was asked to regularly follow up with the surgeons. LBS is a rare patho-clinical entity with a need for standardized diagnostic and treatment regimen. Further case reporting and studies are required to understand the disease in more depth.

## Introduction

Limy Bile Syndrome (LBS) is a rare complication of chronic cholecystitis in which the gallbladder is completely occupied by a thick, tenacious, radio-opaque, and abnormal bile secretion [1]. The presence of limy bile in the hepatobiliary system is one of the rarest causes of obstructive jaundice [2]. Although the bile secretions may have variable consistency and constituents, the most common material is calcium carbonate, followed by bilirubin and phosphates. It is characteristically liquid or semi-liquid in consistency. Endocrine disturbances like hyperparathyroidism have also been reported to be associated with LBS. Double localization is a condition in which this thick and tenacious bile secretion occupies the gallbladder and biliary tree. LBS patients most commonly present with recurrent episodes of upper abdominal pain, nausea, vomiting, and low-grade fever. The term "Limy Bile Syndrome" was coined by Churchman in the year 1911. He is regarded as the first doctor to describe this pathology of the gallbladder. Following this discovery, almost 300 clinical cases of LBS were reported. Knutson in 1933, first described the inorganic composition of limy bile. Subsequently, Volksmann termed the disease as Milk of Calcium Bile disease in 1929. This name does not hold true in all clinical cases of LBS. Therefore, it is not used commonly in the medical literature [3]. Many speculations have been made for the formation of limy bile, like obstruction of the cystic duct, abnormal pH in the gallbladder, and abnormal spontaneous regression of the biliary tract [4]. 

LBS is seen more in females than males and in patients of age 40 years and above. There are no strict guidelines given by any medical body for the management of LBS but the survival rate of patients after cholecystectomy is almost 100%. Surgeons often employ routinely used diagnostic modalities like ultrasonography, hepatobiliary iminodiacetic acid-cholecystokinin (HIDA-CCK) scan, and computer tomography scan for the diagnosis of this condition. There is little data for the prevention of recurrence of limy bile collection in the biliary tree. Ultrasonography reveals a radio-opaque gallbladder. It may invariably be associated with cholelithiasis. Liver function tests suggest an obstructive (surgical) type of jaundice by virtue of elevated alkaline phosphatase enzyme. A complete hemogram reveals increased leukocyte count. The definitive treatment for LBS is laparoscopic cholecystectomy with regular post-operative follow-ups [5]. A combination of endoscopic sphincterotomy and laparoscopic cholecystectomy can be highly effective. Medical management is almost never preferred with only ursodeoxycholic acid (UDCA) agents being prescribed post-operatively. This class of drugs act by stimulating impaired biliary secretion and protecting injured cholangiocytes against harmful toxins in bile. [6].

## Case presentation

A 40-year-old female came with complaints of recurrent pain in the upper abdomen for two years, fatigue for two months, and yellowish discoloration of the eyes for 15 days. The patient had no improvement in symptoms for the last two years, no previous history of trauma over abdomen, black tarry stools, blood in vomitus, gallstones, and blood transfusions.

On examination, all her vital signs were normal except for her heart rate, which was 104 per minute (tachycardia). Murphy’s sign was positive because of mild tenderness at Murphy’s point, which exacerbated on taking a deep breath. Mild jaundice was also noted on examination. Laboratory findings revealed elevated levels of bilirubin and hepatobiliary enzymes. Total bilirubin was 2.0 mg/dL; aspartate aminotransferase (AST) was found to be 64 IU/L, alanine aminotransferase (ALT) was 68 IU/L; and alkaline phosphatase (ALP), 600 IU/dL. Values of all laboratory investigations have been summarized in Table 1.

**Table 1:** Laboratory investigations. Total leucocyte count, total serum bilirubin, AST, ALT, and ALP are found to be elevated. Raised ALP suggestive of obstructive jaundice. * per cubic millimeters, ** milligram per decilitre, *** International Units per litre. References for normal values taken from Gowda S et. al. [7] AST: aspartate transaminase; ALT: alanine transaminase; ALP: alkaline phosphatase

Sr. No.	Laboratory Investigation	Observed Values	Normal Values
1	Total leucocyte count	13,000/mm^3^*	4,500-11,000/mm^3^
2	Total serum bilirubin count	2 mg/dL**	0.0-1.4 mg/dL
3	Serum AST	64 IU/L***	0-35 IU/L
4	Serum ALT	68 IU/L	7-56 IU/L
5	Serum ALP	600 IU/L	41-133 IU/L

**Figure 1:**
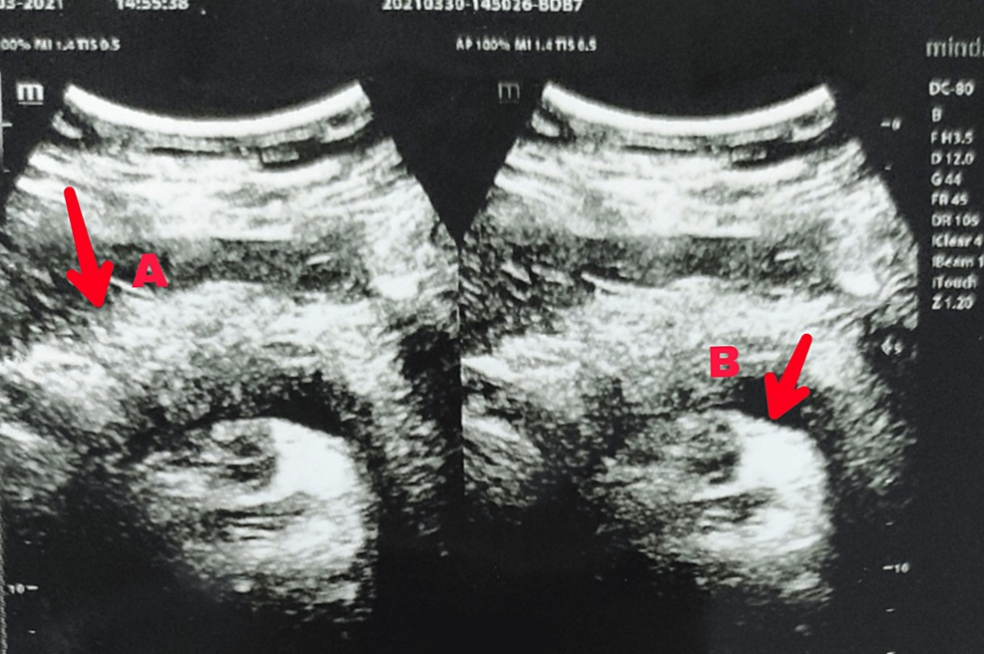
Abdominal ultrasound scan shows a thickened wall of gall bladder (A) and hyperechoic cystic cavity (B) filled with sludge-like fluid. Liver appears to be pale.

**Figure 2:**
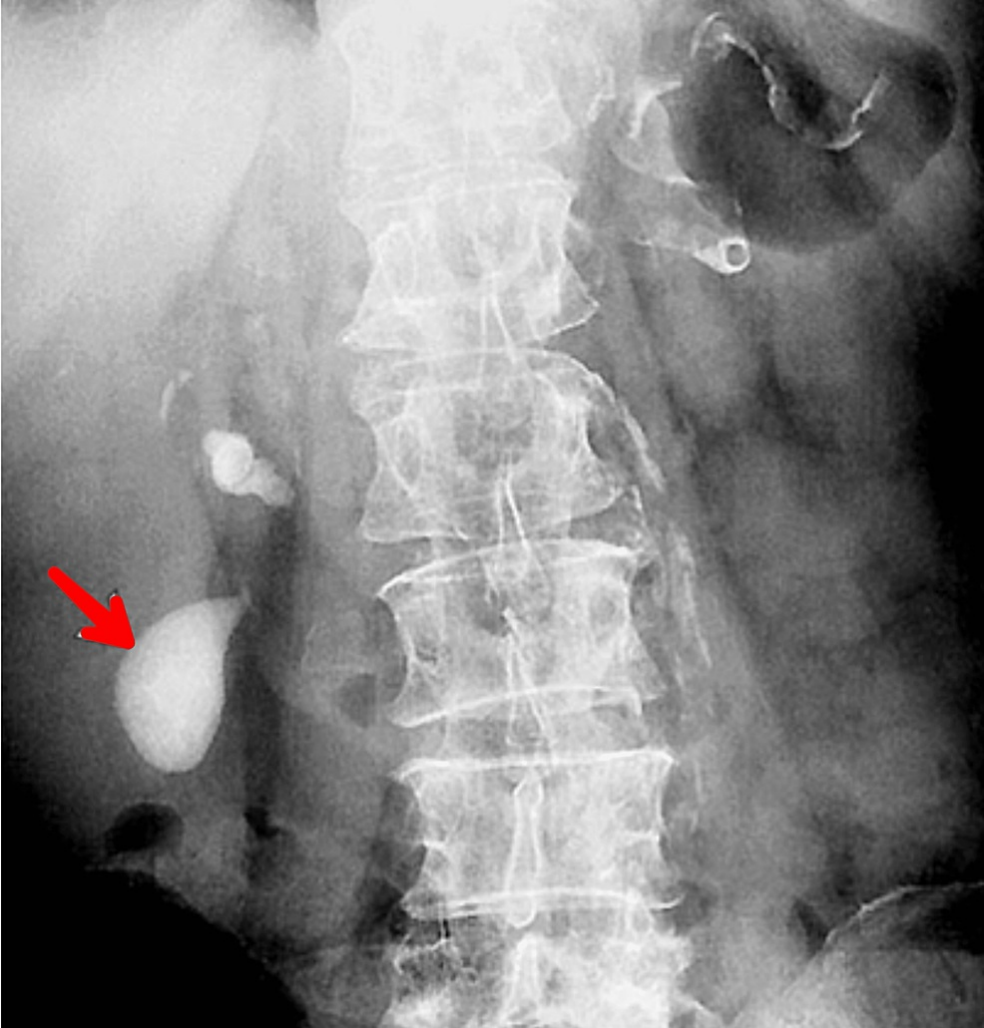
Abdominal x-ray showing a radio-opaque collection of secretions in the gallbladder.

**Figure 3:**
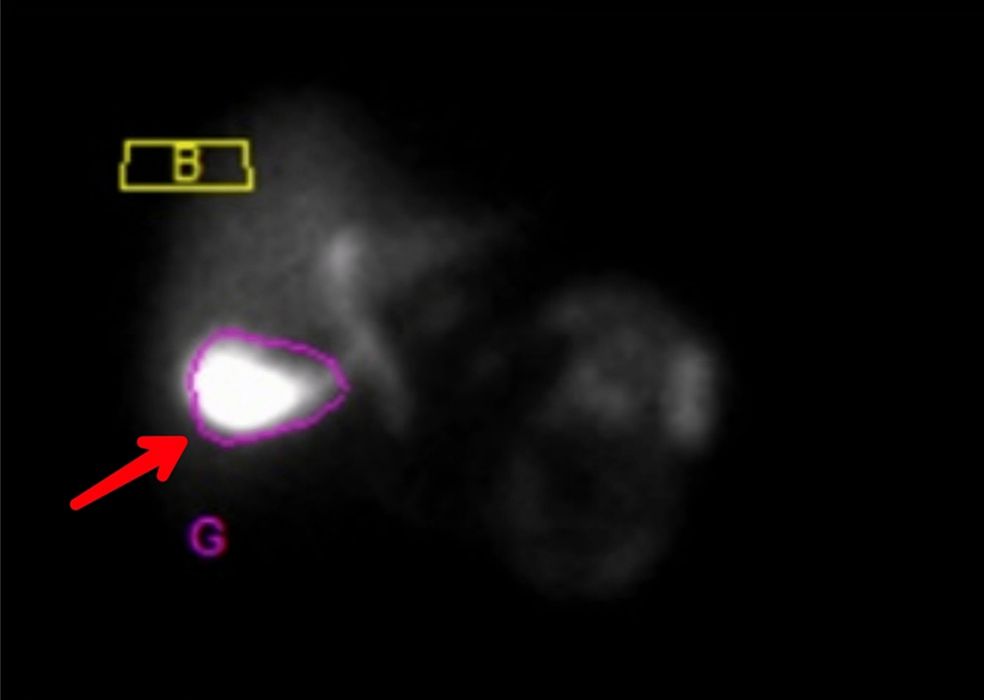
HIDA-CCK scan shows reduced uptake of dye by the hepatobiliary tissues (G) indicating a reduced ejection fraction. HIDA-CCK: hepatobiliary iminodiacetic acid-cholecystokinin

A complete hemogram suggested leukocytosis. The patient was non-diabetic and had no associated comorbidities. A preliminary ultrasound report revealed a thickened gallbladder wall and a hyperechoic cystic cavity, which can be seen in Figure 1. The upper GI endoscopy was uneventful and normal. Subsequent endoscopy revealed a thickened gallbladder with sludge-like secretions inside it. Since ALP was raised, magnetic resonance cholangiopancreaticography (MRCP) and abdominal x-ray were done. The x-ray revealed a radio-opaque gallbladder (Figure 2). HIDA-CCK scan was done to see the contractile capacity of the affected gallbladder (Figure 3). It suggested a gallbladder ejection fraction of 5%.

**Figure 4:**
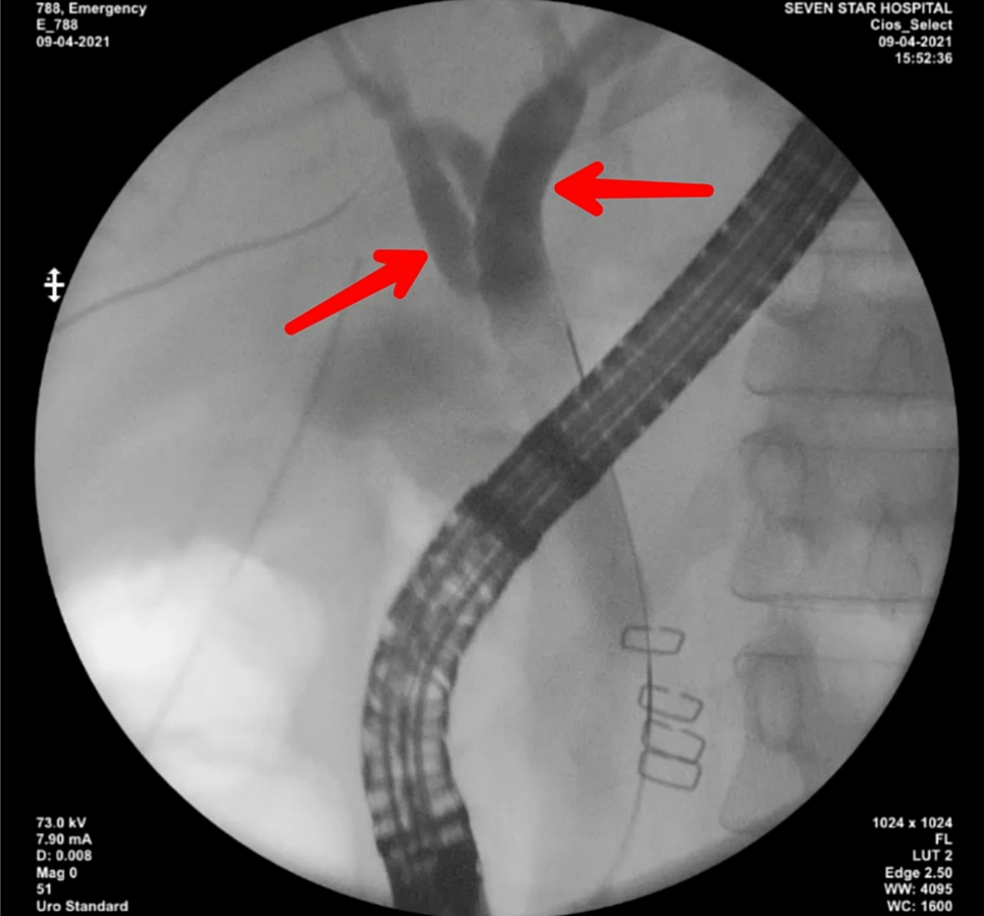
ERCP in LBS shows the CBD and CHD completely filled with thick limy bile. ERCP: endoscopic retrograde cholangiopancreaticography; CBD: common bile duct; CHD: common hepatic duct; LBS: Limy Bile Syndrome

**Figure 5:**
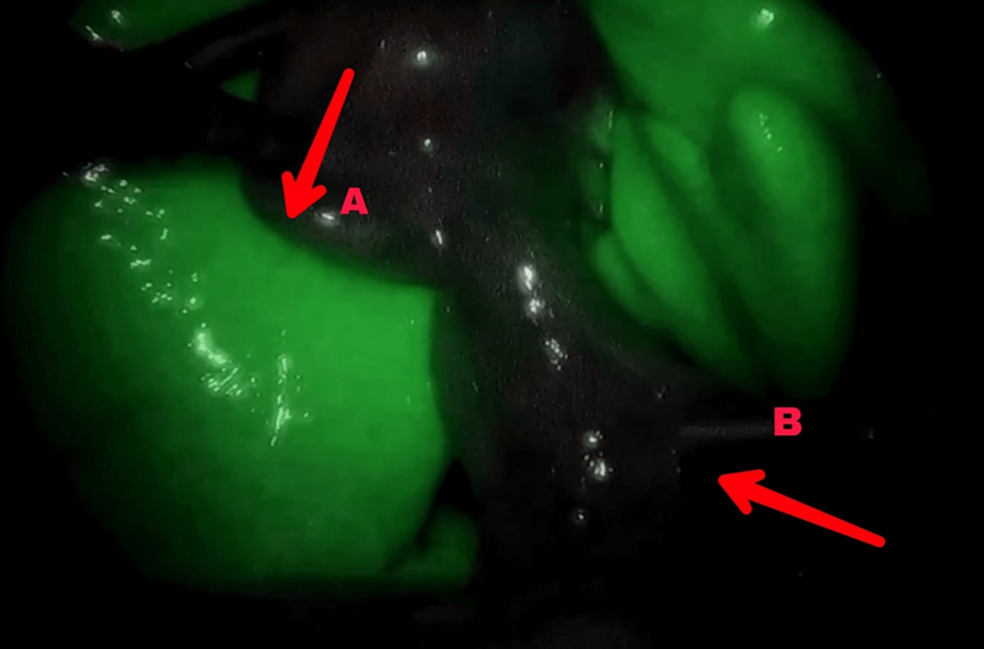
Endoscopic view (assisted with NIR) of ICG-stained hepatobiliary tracts shows a non-illuminated gall bladder (A) and illuminated liver tissue (B) ICG: indocyanine green; NIR: near-infrared imaging

**Figure 6:**
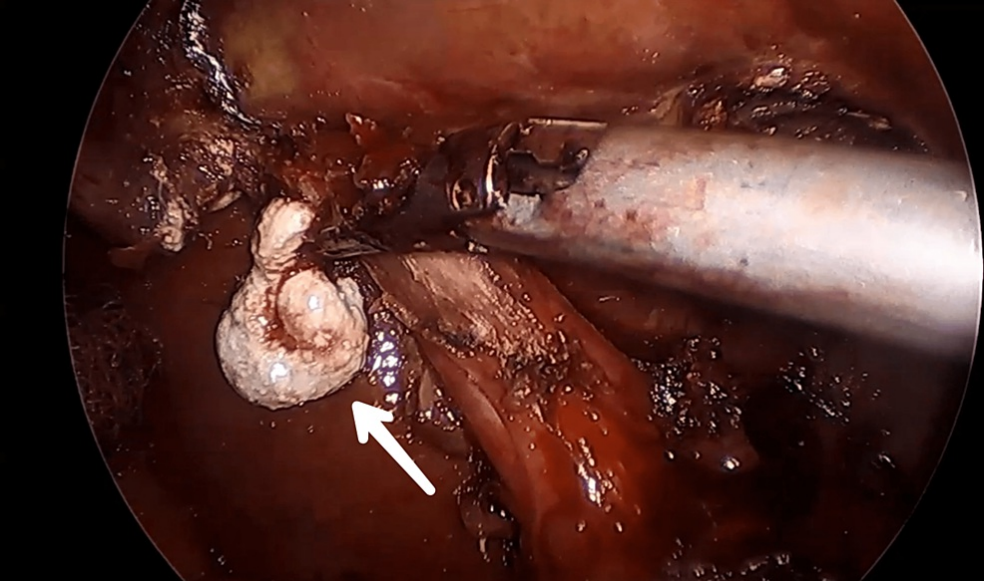
Endoscopic view showing limy bile coming out of gallbladder, seen as thick, white secretion.

After the preliminary diagnostic modalities, endoscopic retrograde cholangiopancreaticography (ERCP) was done. Ample amounts of thick, pasty, limy bile secretions were drained by flushing and sweeping of biliary balloon in the distal common hepatic duct as seen in Figure 4. Consent was obtained from the patient for surgical interventions. Papillotomy was done to prevent future recurrent episodes of LBS. An elective uneventful cholecystectomy was done the next day. It was assisted with near-infrared imaging (NIR) under indocyanine green (ICG) dye. The gallbladder appeared to be completely dark. There was no visualization of the common hepatic duct and common bile duct. Only the liver was illuminated green due to focal accumulation of ICG dye as seen in Figure 5. The cystic duct appeared long with plenty of thick, tenacious limy bile. The pathological secretions were removed by milking technique. The thick, white pasty substance could be seen coming out of the gallbladder under endoscopic view as seen in Figure 6. The resected gallbladder was sent for histopathological examination for any evidence of malignancy. The report was suggestive of chronic cholecystitis without any traces of malignancy. 

The patient was advised to take UDCA preparation for four weeks post-operatively. Her parathyroid hormones, lipid profile, and thyroid status were normal and the patient was stable. Limy bile was an intraoperative surprise and, thus, all causes of LBS were investigated. 

Follow-up was advised to the patient after six months of the procedure. A complete evacuation of limy bile from the gall bladder with no evidence of recurrence was seen. The patient was regularly followed up for the next nine months by doing liver function tests and ultrasonography of the abdomen to rule out any suspicion of recurrence. MRCP was advised one year after cholecystectomy. 

## Discussion

LBS is a sporadic complication of chronic cholecystitis in which the gallbladder is filled with thick, tenacious, radio-opaque, abnormal bile secretions [1]. The formation of limy bile can be attributed to many reasons, most commonly due to obstruction of the cystic duct. It has been hypothesized that patients producing limy bile have abnormal metabolism of calcium and abnormal pH of bile. Calcium can crystallize only at a pH of more than 6.6. Normal biliary pH is over 8. As the pH is always over 6.6, this theory seems unlikely. Another theory suggested that calcium enters bile by the means of passive diffusion instead of active transportation across membranes. A small amount of calcium is absorbed from the gall bladder during the concentration of bile. Bile-salt complexes make up the majority of the calcium in bile. Bile salts increase the solubility of insoluble calcium salts at normal bile pH values. It is possible that some dysfunctional gallbladders may not generate these soluble complexes as easily, leading to the precipitation of limy bile. UDCA is a routinely used agent for therapeutic cholestatic liver disease. Many mechanisms of actions of UDCA have been tested and described aiming at controlling any pathological events in cholestatic liver disorders. UDCA protects injured biliary cells from the toxins in bile. It is also known to stimulate impaired biliary secretions from hepatocytes. UDCA promotes detoxification of hydrophobic bile acids and inhibits early apoptosis of liver cells. Although UDCA has no effect on phospholipid metabolism, it is known to reduce cholesterol secretion in bile. This reduces the chances of the formation of limy bile [8,9].

## Conclusions

In this case, a female patient presented with chief complaints of upper abdominal pain, fatigue, and yellowish discoloration of the eyes. Complete blood count, USG, MRCP, and HIDA-CCK scan pointed to the diagnosis of LBS. The case is quite unique because very few cases of LBS have been identified and treated.

This case describes the unusual manifestation and diagnostic findings of LBS. LBS is a disease not known well in medical sciences. The etiology is unknown with many theories of pathophysiology put forward. Obstruction of the biliary tree is one of the most accepted mechanisms of the disease. LBS is known to have a good prognosis after cholecystectomy is done in the early phases of progression. To prevent further recurrence of LBS, drugs like UDCA play an important role. The lack of any strict regimen of treatment can be mainly attributed to the few cases detected and diagnosed. Further scope for research is a preliminary need for preventing the occurrence of LBS in the first place.
